# Access, Safety, and Barriers in Adoption of Emergency Laparoscopy Surgery for Trauma Patients in a Low-Resource Setting

**DOI:** 10.1055/s-0043-1761951

**Published:** 2023-03-03

**Authors:** Rahul Kumar, Arpan Mishra, Harikrishna Damde, Prasant Yadav, Sanjay Kumar Yadav

**Affiliations:** 1 Department of Surgery, Netaji Subhash Chandra Bose Medical College, Jabalpur, Madhya Pradesh, India

**Keywords:** emergency laparoscopy, blunt trauma abdomen, safety, barriers

## Abstract

**Introduction** This study analyzes barriers to the adoption of emergency laparoscopy (EL), safety, and accessibility in a low-resource setting of a low- and middle-income country (LMIC).

**Methods** In this prospective observational study, patients with blunt trauma abdomen (BTA) who required exploration were included and divided into two groups—open exploration (open surgery [OSx]) and laparoscopic exploration (laparoscopic surgery [LSx]). Data were compiled and analyzed.

**Results** Out of 94 BTA patients, 66 required exploration, and the rest were managed conservatively. Out of 66 patients, 42 were in OSx and 24 were in LSx, reason for not selecting LSx was the surgeon's preference for OSx in 26 patients and the lack of availability of operation theater (OT) slots in 16 patients. LSx even after indication was less likely if patients had preoperative evidence of perforation peritonitis.

**Conclusion** Lack of resources (OT availability and trained personnel) are barriers to the adoption of emergency LSx in low-resource settings.

Despite the significant improvements in emergency laparoscopy (EL) for trauma patients in developed countries, the adoption of laparoscopy in low- and middle-income countries (LMICs) has been sporadic and minimal as it requires enhanced surgical expertise. 1 Multiple intra-abdominal injuries make laparoscopic exploration more difficult, and advanced laparoscopic skills are required. Other barriers could be a lack of capacity and affordable availability. 2

The overall aim of this study was to analyze barriers to the adoption of EL, safety, and accessibility in a low-resource setting of an LMIC.

## Methods

This prospective study was conducted at the Department of General Surgery, Netaji Subhas Chandra Bose Medical College, Jabalpur, India from September 1, 2018, to September 31, 2020, after approval from Institutional Ethics Committee. Patients requiring emergency abdominal exploration who were hemodynamically stable with the following criteria were included:

1. Focused assessment sonography in trauma positive.
2. Signs of peritonitis, evidence of perforation.
3. High index of suspicion of abdominal injury based on mechanism of injury and signs of external injury without obvious signs of peritonitis and computed tomography (CT) scan not feasible due to financial constraints.

Patients with the following criteria were excluded:

1. Hemodynamic instability.
2. COVID-19 suspect/positive.
3. Children and pregnant women.
4. Patients who were not fit for general anesthesia.
5. Patients who did not give consent.

### Definitions

Patients eligible for laparoscopy were required to be hemodynamically stable (systolic blood pressure more than 90 mm Hg). Negative laparoscopy was defined as a procedure when no injury was detected. Therapeutic laparoscopy was defined as a procedure when surgical intervention was successful laparoscopically. Nontherapeutic laparoscopy was labeled when surgical intervention had no impact on the patient's outcome. Laparotomy was similarly classified as either therapeutic or nontherapeutic.

**Fig. 1:**
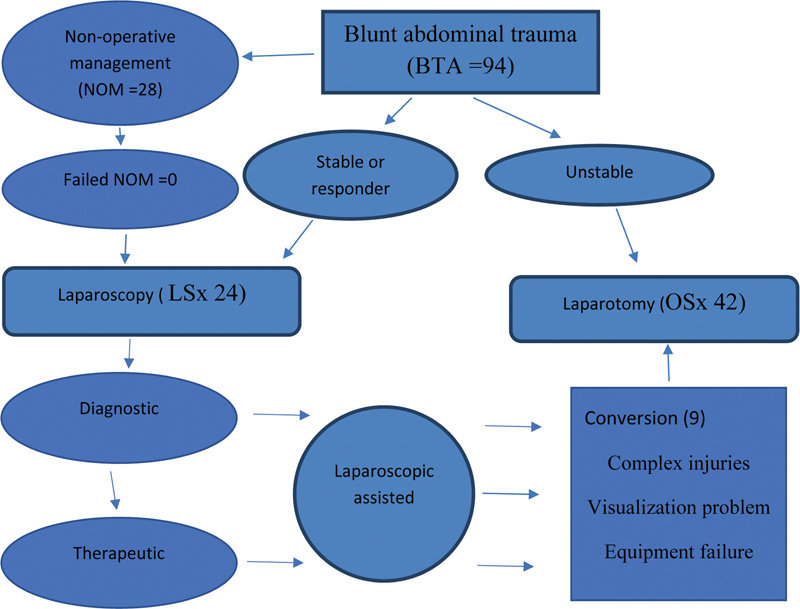
Management protocol. LSx, laparoscopic surgery; OSx, open surgery.

The management protocol is depicted in Fig. 1 .

### Statistical Analysis

Mean, median, range, and frequencies were reported as descriptive statistics. They were compared using the chi-square test and the Student's *t* -test for qualitative and quantitative parameters as appropriate. A *p* -value of <0.05 was considered significant. All statistical analyses were done using SPSS V16.

## Results

A total of 94 blunt trauma abdomen (BTA) patients were seen in our emergency department during the study period. Out of these, 66 patients required abdominal exploration, and the rest 28 were managed conservatively. Out of 66 patients who underwent exploration, 42 were in the open surgery (OSx) group and 24 were in the laparoscopic surgery (LSx) group. In the OSx group, six patients (14.28%) had negative laparotomy; in the LSx group, nine patients were converted to open and had no missed injuries. The two groups' basic demographic profile, clinical presentation, intraoperative findings, surgical procedure, and postoperative outcome are depicted in Table 1 .

**Table 1:** Organ involvement and hemoglobin comparison of both the groups

		LSx	OSx	*p* -Value ( *t* test)	
Male:female		23:1	36:6	NA	
Hemoglobin (g/dL)	<5	0	4	0.12	NS
	>5	24	38	0.73	NS
FAST and X-ray	Bowel perforation	3	26	0.001	S
	Spleen injury	9	8	0.76	NS
	Liver injury	6	2	0.2	NS
	Other	6	6	0.9	NS
CT	Bowel perforation	6	24	0.001	S
	Spleen injury	15	8	0.42	NS
	Liver injury	3	2	0.12	NS
	Other	3	8	0.2	NS
OT finding	Bowel perforation	6	24	0.001	S
	Spleen injury	15	8	0.42	NS
	Liver injury	3	2	0.12	NS
	Other	3	8	0.2	NS

Abbreviations: CT, computed tomography; FAST, focused assessment sonography in trauma; LSx, laparoscopic surgery; NA, not available; NS, not significant; OSx, open surgery; OT, operation theater; S, significant.

Patients with low hemoglobin levels are managed with OSx and there was a good co-relation between CT and operation theater (OT) findings. Intensive care unit and hospital stay were found to be statistically significant between OSx and LSx ( Table 2 ). From the total 42 open explorations, the main reason identified was the lack of OT in 16 cases, of which 6 were operated in routine hours due to a long routine OT list and 10 in emergency hours due to more than 3 laparotomy cases in a single night in 8 cases and technical failure was encountered in 2 cases ( Table 3 ).

**Table 2:** Postoperative outcome

		LSx	OSx	*p* -Value ( *t* test)	
**1**	Postoperative fever	37.5	53.0	0.1245	NS
**2**	Postoperative pain	0.87	4.14	1.79	NS
**3**	Wound infection	1	2.57	6.2132	NS
**4**	Full oral diet (in d)	3.25	7.69	9.884	NS
**5**	Mobilization (in d)	3.8	5.33	3.311	NS
**6**	Hospital stay (in d)	6.75	10.09	0.0018	S
**7**	ICU stay (in d)	1.625	2.33	0.0051	S
**8**	Operative time (induction to reversal in hours)	3.7	5.3	0.8	NS

Abbreviations: ICU, Intensive care unit; LSx, laparoscopic surgery; NS, not significant; OSx, open surgery; S, significant.

**Table 3:** Reason for not selecting LSx ( *n* = 42)

1	Surgeon's preference (26)	Perforation peritonitis (20) History of previous surgery (6)
2	Lack of availability of laparoscopy operation room (16)	In routine hours (6) In emergency hours (10) More than 3 laparotomy cases in single night (8) Technical failure (2)

Abbreviation: LSx, laparoscopic surgery.

## Discussion

Our study reveals that even when laparoscopic equipment is available, the availability of OT was a major barrier in adopting EL for BTA patients apart from the lack of trained personnel. Dissemination of technology to LMICs will not have an impact on clinical outcomes unless it is accompanied by resources to implement these technologies. A similar study by Choy et al 1 identified three overarching barriers to the uptake of laparoscopy in LMICs—(1) the organizational structure for funding laparoscopic procedures, (2) the hierarchical nature of the local surgical culture, and (3) the expertise and skills associated with a change in practice.

Many studies 3 have reported cost as one of the significant barriers for patients because cheaper alternatives are available, and our study did not have a cost factor as all services are freely available. Apart from the availability of OT, this study also identified two other important barriers that are scarcely reported in the literature. Surgeons' preference for open laparotomies is the second major barrier. We cannot force anyone to perform LSx; patient safety should be the most important aspect in selecting a procedure. However, due to the heavy operation load, senior surgeons do not find the time to train themselves, leading to very slow adoption. They might have opted for laparoscopy if they had local access to experienced laparoscopic trainers. So, this lack of local access to experienced laparoscopic trainers and/or lack of time to train themselves are barriers that limit the use of widespread laparoscopy in trauma patients. 4 A total of 21 nontherapeutic laparotomies were performed during the study period which could have been avoided. The success rate of therapeutic laparoscopy for trauma patients is less (∼ 20%), but patients undergoing LSx have significantly shorter stays and early recovery. 5 It should not be expected that surgeons be able to perform the laparoscopic repair of bowel injuries, but we should adopt these new techniques to stay current in their field and to improve patient outcomes. As can be seen from our data that nine patients required conversion to OSx after laparoscopy.

As the world population is growing by 1.18% per year, reaching 8.5 billion in 2030, and increasing further to 9.7 billion in 2050. What is more concerning is the fact that half of the world's population growth will be in just nine countries (India, Nigeria, Pakistan, Democratic Republic of the Congo, Ethiopia, United Republic of Tanzania, United States, Indonesia, and Uganda) and all of them being LMICs except the United States. 6 This heavy burden puts limitations in learning new skills for practicing surgeons, as we have mentioned that only two faculties are trained. This training is based on exposure to laparoscopy during postgraduation, which is inconsistent and informal. Alternative training opportunities are limited by availability and cost. Another study has also pointed out these findings. 7

A major limitation of our study is that it is a single-center study with a small number of patients.

## Conclusion

Lack of resources (OT availability and trained personnel) are barriers to the adoption of emergency LSx in low-resource settings.
